# The Metric System in Machine Shops

**Published:** 1881-02

**Authors:** 


### THE METRIC SYSTEM IN MACHINE SHOPS.

In the November number of the *Journal of the Franklin Institute* is a strong article by Mr. Coleman Sellers, showing why the metric system should *not* be introduced into American machine shops. His arguments are conclusive, and we recommend them to the perusal of the metric fanatics. They apply in a great degree to dispensing pharmacy, as well as to the manufacture of machines. His comments on the conflict of meteorological systems are acute, and sufficiently indicate the difficulties which stand in the way of any harmonious action.

There is no doubt but that the more closely the metric system is scanned and tested by practical men, the less does its introduction recommend itself. Shall it not be dropped altogether?

